# Citrulline and ADI-PEG20 reduce inflammation in a juvenile porcine model of acute endotoxemia

**DOI:** 10.3389/fimmu.2024.1400574

**Published:** 2024-08-08

**Authors:** Caitlin Vonderohe, Barbara Stoll, Inka Didelija, Trung Nguyen, Mahmoud Mohammad, Yava Jones-Hall, Miguel A. Cruz, Juan Marini, Douglas Burrin

**Affiliations:** ^1^ USDA-ARS Children’s Nutrition Research Center, Department of Pediatrics, Baylor College of Medicine, Houston, TX, United States; ^2^ Pediatric Critical Care Medicine, Department of Pediatrics, Baylor College of Medicine, Houston, TX, United States; ^3^ Center for Translational Research on Inflammatory Diseases (CTRID), Michael E. DeBakey Veteran Administration Medical Center, Houston, TX, United States; ^4^ Department of Pathobiology, Texas A&M College of Veterinary Medicine and Biomedical Science, College Station, TX, United States; ^5^ Department of Medicine, Baylor College of Medicine, Houston, TX, United States

**Keywords:** sepsis, arginine, NO, ADI-PEG20, citrulline

## Abstract

**Background:**

Arginine is a conditionally essential amino acid that is depleted in critically ill or surgical patients. In pediatric and adult patients, sepsis results in an arginine-deficient state, and the depletion of plasma arginine is associated with greater mortality. However, direct supplementation of arginine can result in the excessive production of nitric oxide (NO), which can contribute to the hypotension and macrovascular hypo-reactivity observed in septic shock. Pegylated arginine deiminase (ADI-PEG20, pegargiminase) reduces plasma arginine and generates citrulline that can be transported intracellularly to generate local arginine and NO, without resulting in hypotension, while maintaining microvascular patency. The objective of this study was to assess the efficacy of ADI-PEG20 with and without supplemental intravenous citrulline in mitigating hypovolemic shock, maintaining tissue levels of arginine, and reducing systemic inflammation in an endotoxemic pediatric pig model.

**Methods:**

Twenty 3-week-old crossbred piglets were implanted with jugular and carotid catheters as well as telemetry devices in the femoral artery to measure blood pressure, body temperature, heart rate, and respiration rate. The piglets were assigned to one of three treatments before undergoing a 5 h lipopolysaccharide (LPS) infusion protocol. Twenty-four hours before LPS infusion, control pigs (LPS; n=6) received saline, ADI-PEG20 pigs (n=7) received an injection of ADI-PEG20, and seven pigs (ADI-PEG20 + CIT pigs [n=7]) received ADI-PEG20 and 250 mg/kg citrulline intravenously. Pigs were monitored throughout LPS infusion and tissue was harvested at the end of the protocol.

**Results:**

Plasma arginine levels decreased and remained low in ADI-PEG20 + CIT and ADI-PEG20 pigs compared with LPS pigs but tissue arginine levels in the liver and kidney were similar across all treatments. Mean arterial pressure in all groups decreased from 90 mmHg to 60 mmHg within 1 h of LPS infusion but there were no significant differences between treatment groups. ADI-PEG20 and ADI-PEG20 + CIT pigs had less CD45+ infiltrate in the liver and lung and lower levels of pro-inflammatory cytokines in the plasma.

**Conclusion:**

ADI-PEG20 and citrulline supplementation failed to ameliorate the hypotension associated with acute endotoxic sepsis in pigs but reduced systemic and local inflammation in the lung and liver.

## 1 Introduction

Sepsis is the leading cause of mortality and critical illness in adults and children (1–3). More than 72,000 children are hospitalized annually in the United States for sepsis, and the condition has a mortality rate of 25% and annual cost of $4.8 billion (1, 4, 5). Pediatric sepsis and multiple organ dysfunction (MODS) have been largely understudied, despite being a primary cause of morbidity and mortality in critical illness. Refractory shock (6), a clinical condition wherein circulatory failure results in systemic hypoxia and severe disruptions to body processes, remains a common cause of MODS and early death in pediatric sepsis (7). In refractory shock, patients acutely fail to respond to resuscitation with fluids, inotropes, and vasopressors after septic shock, indicating a need for alternative strategies to manage and treat sepsis and MODS (8, 9).

Arginine is a conditionally essential amino acid in infancy for protein synthesis but is also the precursor for multiple metabolites that are essential for organ function, including nitric oxide (NO), urea, creatine, proline, and glutamate. Arginine plays a central role in energy metabolism, ammonia detoxification, and the regulation of blood pressure and immunity (10). Arginine’s central role in NO production makes it a pharmaceutical and nutritional inhibitory target to improve outcomes in cases of shock and sepsis. However, thus far, attempts to inhibit NO production in shock and sepsis have yielded mixed results in animal and clinical trials (11–15).

The pervasive nature of arginine in the maintenance of such diverse processes as nitrogen metabolism, cell signaling, and blood pressure homeostasis means that the regulation of arginine availability can be complex (10). In healthy subjects, approximately 40% of dietary arginine is metabolized during first pass metabolism (16–18). Arginase is the primary pathway for arginine disposal; it produces ornithine and urea (18). There are two isoforms of arginase: arginase I (liver type) is cytosolic, whereas arginase II (kidney type) is mitochondrial (10, 19). Arginase activity is critical to the metabolism of arginine during sepsis and inflammation because both of these isoforms are present in macrophages and endothelial cells (20–22).

Macrophages metabolize arginine to ornithine, which can result in the production of polyamines, which may facilitate tissue healing but can also be co-opted by pathogens to improve survivability in the face of the immune system or adversely impact the host response to pathogens (23, 24). Intracellular arginine availability is determined as a net combined metabolism from circulating arginine, utilization among the completing pathways, the citrulline recycling pathway, and transporter activity (25, 26). In endothelial cells, arginine metabolism to NO via NO synthase leads to vasodilation and hypotension and under conditions of sepsis can lead to organ failure and mortality (27). Additionally, when arginine is metabolized, NO synthase produces superoxide and then peroxynitrite, a powerful oxidant that can lead to organ damage (28).

Most normal healthy cells in the body have the capacity to take up citrulline from the blood and convert it to arginine via the enzymes arginosuccinate lyase (ASL) and arginosuccinate synthase (ASS) (29, 30). Citrulline supplementation increases arginine availability more effectively than arginine supplementation alone in healthy conditions because it has a longer plasma half-life and can be converted to arginine as needed in specific tissues; arginine is more likely to be metabolized before reaching specific arginine-deficient tissues (31). Briefly, the rate limiting step in the conversion of citrulline to arginine is catalyzed by ASS (32). Arginosuccinate synthetase activity is inhibited by metabolite (Cys132) production of NO, meaning that in cases of inflammation or NO production, arginine synthesis from citrulline is limited (33). This means that citrulline supplementation is less likely to result in NO overproduction than arginine (34–36). Although limited work has been carried out to assess the impact of citrulline supplementation in cases of inflammation or sepsis in the clinic, *in vitro* work conducted by Reizine et al. (37) and Breuillard (38) showed that citrulline can modulate macrophage and T-cell activity (37, 38). A recent clinical trial suggested that citrulline supplementation in patients with non-alcoholic fatty liver disease reduces inflammatory cytokines; however, a more recent clinical trial showed that enteral citrulline supplementation did not have an impact on sepsis scores in critically ill patients (39, 40). In a rodent model of endotoxemia, citrulline was more effective than arginine in reducing intestinal microcirculatory dysfunction (41). Citrulline also functions as an antioxidant that may reduce hydroxyl radical formation and therefore reduce oxidative tissue damage (42).

Some cancer cell types lack ASS and ASL and cannot produce arginine, making them dependent on free blood arginine for protein synthesis and tumorigenic growth (43). Arginine deiminase is a bacterial enzyme that reduces the imino group of free arginine to produce citrulline and ammonia. ADI-PEG20 is arginine deiminase that has been pegylated to increase its half-life to approximately 10 days and reduce immunogenicity. ADI-PEG20 has been successfully used in cancer patients to reduce tumor size (44, 45). Treatment with ADI-PEG20 drastically reduces circulating free arginine (<2 μmol/L), which results in the death and regression of tumor cells (46, 47).

**Figure 1:**
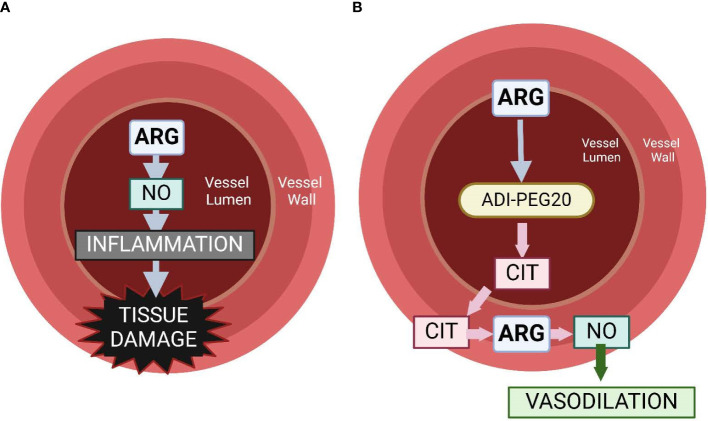
Graphical representation of the hypothesis of the physiology associated with intravascular arginine **(A)** vs. the use of ADI-PEG20 **(B)**. We hypothesize that the use of ADI-PEG20 in an endotoxemia model will effectively remove arginine from the lumen of the vasculature, preventing it from becoming intralumenal NO, which can be a potent pro-inflammatory cytokine. ADI-PEG20 catalyzes the conversion of arginine to citrulline. Citrulline is transported out of the vasculature into the vessel wall or into the tissue where it can be converted into arginine, and then NO acts as a vasodilator, preventing hypoxic injury to tissue.

Using ADI-PEG20 during periods of high arginase activity (as in sepsis) preempts the conversion of plasma arginine to ornithine and urea but spares intracellular arginine because citrulline can be reused in the citrulline recycling pathway to produce intracellular arginine (30). Marini et al. (30) demonstrated that giving ADIPEG-20 to endotoxemic mice spares tissue function that is dependent on intracellular arginine, while decreasing NO production by approximately 50% (30, 48). The objective of this study was to assess the efficacy of ADI-PEG20 with and without supplemental intravenous citrulline to mitigate hypovolemic shock, maintain tissue levels of arginine, and reduce systemic inflammation in an endotoxemia pediatric pig model. We hypothesize that ADI-PEG20 will maintain blood pressure, reduce tissue-level inflammation, and dramatically reduce plasma arginine, while preserving tissue arginine (**Figure 1**).

## 2 Materials and methods

### 2.1 Animal procedures

All animal procedures were approved by the Baylor College of Medicine Institutional Animal Care and Use Committee. Twenty male and female conventionally raised 3-week-old pigs were obtained from a commercial swine farm. The pigs were transported to the Children’s Nutrition Research Center animal facility 10–12 days before surgery for acclimation, where they were gradually transitioned from milk replacer (NutriStart Liqui-Wean, Milk Specialties Co., Eden Prairie, MN, USA) to solid pelleted feed (Mini Pig Starter Diet 5080, LabDiet, St. Louis, MO, USA).

**Figure 2:**
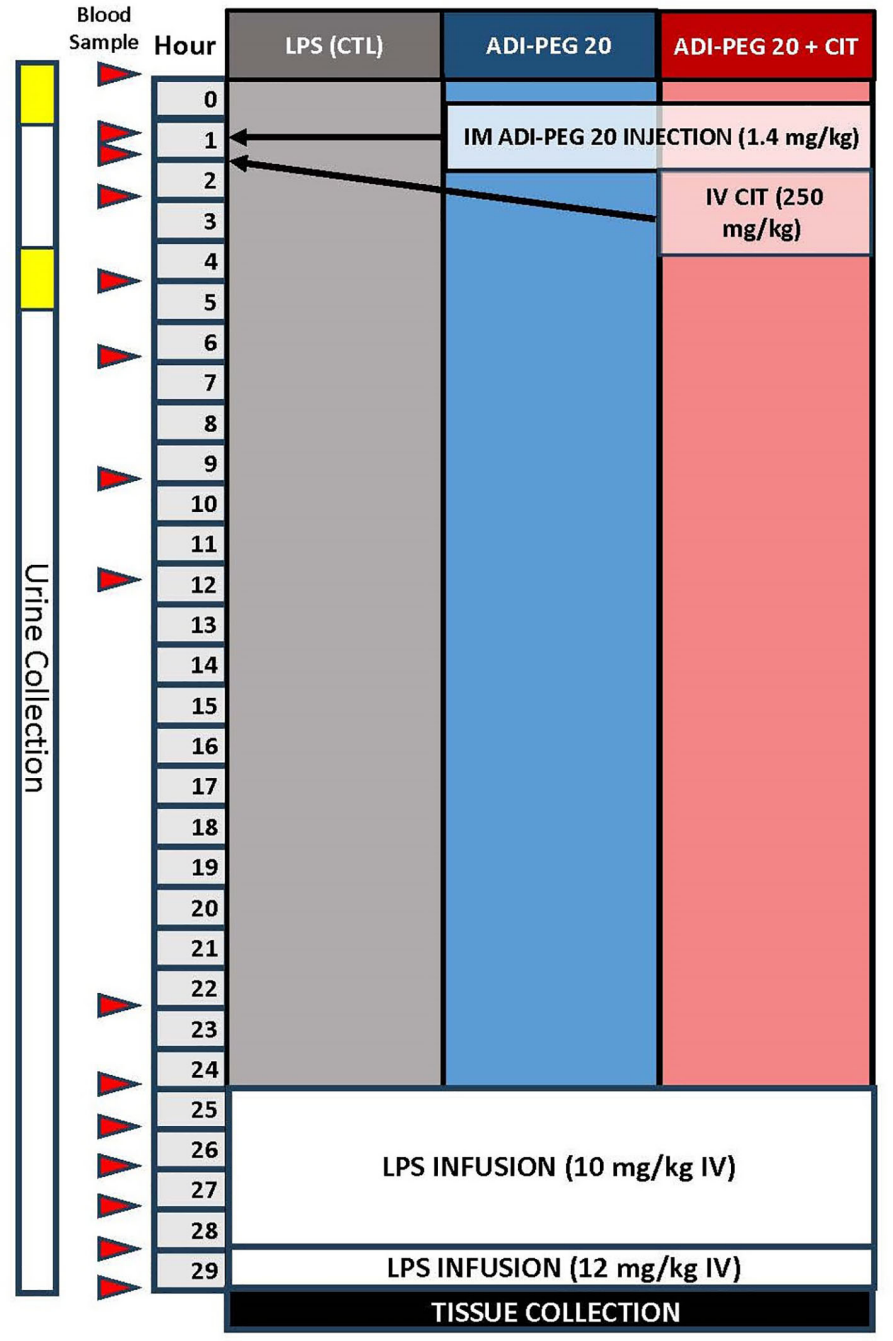
Schematic of the study design and blood sampling protocol relative to lipopolysaccharide infusion, ADIPEG injection, and citrulline bolus.

The pigs were anesthetized using isoflurane, and jugular and carotid catheters were surgically implanted as described previously (49). Additionally, the pigs were implanted with a telemetry probe (M10, Data Science International, St. Paul, MN, USA) in the femoral artery for constant monitoring of blood pressure, temperature, heart rate, and respiration rate. The pigs were allowed to recover from surgery for 2 days before ADI-PEG20 injection and subsequent LPS infusion. The pigs wore harnesses and were attached to swivels to facilitate freedom of movement within stainless steel cages throughout the experiment. A control group (LPS; n=6) underwent surgery but received no additional treatment until the LPS infusion. ADI-PEG20 (1.4 mg/kg ~ 12 IU/kg) was given intramuscularly to LPS + CITRULLINE + ADI-PEG20 (ADI-PEG20+CIT; n=7) and LPS + ADI-PEG20 (ADI-PEG20; n=7) pigs, and sterile saline was injected intramuscularly to the LPS (n=6) pigs 24 h before lipopolysaccharide (LPS) infusion. Citrulline (250 mg/kg) was intravenously infused as a bolus to ADI-PEG20+CIT pigs 30 min after ADI-PEG20 administration. Blood was collected via the carotid catheter before ADI-PEG20 administration and 30 min, 60 min, 2 h, 4 h, 6 h, 9 h, 12 h, and 22 h after ADI-PEG20 administration (**Figure 2**). Urine was collected in clean pans under the cages before and after ADI-PEG20 and citrulline administration from two pigs per treatment (as available before and after the initiation of treatment).

LPS (lyophilized *E. coli* Serotype 0111-B4, Sigma Chemical, St. Louis, MO, USA) was infused (10 μg*kg^-1^*hr^-1^ for 4 h, then 12.5 μg*kg^-1^*hr^-1^for 1 h). Blood was sampled immediately before infusion, then hourly until tissue harvest (**Figure 2**). Blood glucose was measured every 15 min throughout the LPS infusion using a glucometer, and dextrose was infused into the jugular vein to maintain a target arterial blood glucose of >70 mg/dL. An ISTAT system with a CG8+ cartridge (Abaxis, Union City, CA, USA) was used for cage-side analysis of sodium, chloride, bicarbonate, anion gap, pH, partial pressure of carbon dioxide, total carbon dioxide, base excess in the extracellular compartment, hemoglobin, and hematocrit. At the end of the LPS infusion, or when the animal reached early humane euthanasia criteria (central nervous system dysfunction, severe dysglycemia refractory to dextrose infusion, or hypotension refractory to fluid resuscitation defined as mean arterial blood pressure less than 30% from baseline and a heart rate of more than 50% from baseline for more than 3 h) during infusion, animals were euthanized and tissue was collected.

### 2.2 Sample analysis

Plasma and urinary citrulline, arginine, phenylalanine, tyrosine, isoleucine, ornithine, and leucine concentrations were measured by liquid chromatography with tandem mass spectrometry after derivatization using dansyl chloride as described previously (48) and a commercially available internal standard (U-^13^C U-^15^N amino acid mix, Cambridge Isotope, Andover, MA, USA). To measure tissue concentrations of these amino acids, small intestine, liver, lung, skeletal muscle, kidney, and heart tissues were homogenized on ice in Triton X-100 (2 g/L) after the addition of the internal standard. An aliquot of the supernatant was derivatized with dansyl chloride.

Quantitative real-time PCR was performed on samples of small intestine, lung, liver, kidney, and skeletal muscle (longissimus dorsi) tissue. Total RNA was isolated using TRIzol reagent (Invitrogen) and a commercial kit (Qiagen). Total RNA was analyzed for concentration and quality using a spectrophotometer (NanoDrop, Thermo Scientific) and subsequently diluted to 250 ng/μl. Reverse transcription was carried out using a commercial kit (Applied Biosystems). Real-time PCR was performed on a Bio-Rad CFX96 using PowerUp SYBR Green Master Mix (Applied Biosystems). Relative mRNA expression within each tissue was quantified relative to β-actin using the 2-ΔΔCT method (see **Table 1** for primers). Fold change was calculated relative to the mean expression of each gene (ΔCT) in the LPS treatment.

**Table 1:** PCR Primers.

Primer	Forward	Reverse
**β-ACTIN**	GGACCTGACCGACTACCTCA	GCGACGTAGCAGAGCTTCTC
**ARG1**	GGCTGGTCTGCTTGAGAAAC	AGCCAGCTGTTGATTTGCTT
**ARG2**	TACGTCCTGCCCTTCGTATC	CAAGCCAGCTTCCCTTACAG
**ASL**	AGTTCCTGTTCTGGGCTTCG	GCTTCCAGTGCTGTAGGCAT
**ASS**	GCTGGTGTACACGGGTTTCT	CCAGCTCCTCGTTGTAGAGG
**TNFA**	GGCCCAAGGACTCAGATCAT	TGAGGTACAGCCCATCTGTC
**IL6**	TCTGGGTTCAATCAGGAGACC	CTAATCTGCACAGCCTCGAC
**IL1B**	AGGCAGATGGTGTCTGTCATC	AGGATGATGGGCTCTTCTTCAAA
**IL17**	CGGCTGGAGAAAGTGATGGT	CAGAAATGGGGCTGGGCT
**IL-8**	GGCAGTTTTCCTGCTTTCT	CAGTGGGGTCCACTCTCAAT

Plasma cytokine concentrations, interferon gamma (IFN-G), interleukin 1-beta (IL1B), interleukin-6 (IL6), interleukin-8 (IL8), interleukin-10 (IL10), and tumor necrosis factor-alpha (TNFA) were analyzed at the University of Maryland Cytokine Core (Baltimore, MD) lab using a porcine-specific, multiplex assay.

### 2.3 Histology and pathology

At the end of the LPS infusion, or when animals reached early humane euthanasia criteria during infusion, animals were euthanized using a commercially available pentobarbital solution (SomnaSol, Henry-Schein Animal Health, Dublin, OH, USA), necropsied, and tissue damage semi-quantitatively scored by a licensed veterinarian (CEV) blinded to the treatment groups. Tissue was fixed for histopathology in 10% neutral buffered formalin then stained with hematoxylin and eosin (H&E). The H&E slides were then scored by a boarded veterinary pathologist (YJH) blinded to the treatment groups. Additional, unstained slides were deparaffinized in xylene and rehydrated through graded alcohol solutions. Antigen retrieval was performed in a pressure cooker using a pH 6.2 buffer (Diva, Biocare Medical, Pacheco, CA, USA). The procedure was run on an autostainer (intelliPATH FLX, Biocare Medical). The CD45 antibody, clone MAC323, (Biorbyt, Durham, NC, USA) was diluted 1:100 and incubated for 1 h at room temperature. The slides were then incubated for 30 min with a detection reagent (Mach 2 Mouse HRP Polymer, Biocare Medical). DAB chromogen was used to detect sites of antibody-antigen interaction. Hematoxylin was used as the counterstain. A negative tissue control was run by substituting a negative isotype control for the primary antibody.

Slides were scanned at 20× magnification using a Pannoramic Scan II by 3DHistec, and images were analyzed using Visiopharm Software (Version 2022.9 Hoersholm, Denmark) (50–52). Briefly, the tissue to be analyzed was outlined using an algorithm to detect the whole tissue on the digital slide and define it as the region of interest (ROI). For the CD 45 immunohistochemistry, a custom artificial-intelligence-based Visiopharm algorithm was developed to detect the total number of nuclei in the tissue and the nuclei of cells with positive cytoplasmic labeling (CD45 positive cells). The APP then quantified the number of CD45 positive cells/mm2 within the ROI. This quantification was expressed as “CD45+ cells”.

### 2.4 Statistical analysis

Data are presented as mean ± standard error (SE). Data were analyzed using Prism GraphPad 9.2.0. Telemetry, plasma cytokine, plasma amino acid, and iStat data generated from the pigs during the endotoxin infusion were analyzed using a mixed model ANOVA with time and treatment as fixed effects and pig and litter as random effects. Histology, pathology, gene expression, and tissue amino acid levels were tested for normal distribution and analyzed using a two-way ANOVA with main effects of treatment and litter. Differences between means were considered significant with *P*-values < 0.05.

## 4 Results

### 4.1 Endotoxemia outcomes

Of the 20 pigs that underwent the endotoxin infusion protocol, five were euthanized before the end of the 5 h protocol (n=2 LPS; n=2 ADI-PEG20; n=1 ADI-PEG20 + CIT). All pigs were supported by a 0.8mL*kg^-1^*hr^-1^ infusion of 50% dextrose that was adjusted as needed based on blood glucose measures every 15 min throughout the endotoxin infusion. Additionally, when mean arterial pressure (MAP) fell below 60 mmHg for longer than 15 min, a 20 mL/kg bolus of sterile 0.7% saline was administered. This intervention was applied to three pigs (one of each treatment).

**Figure 3:**
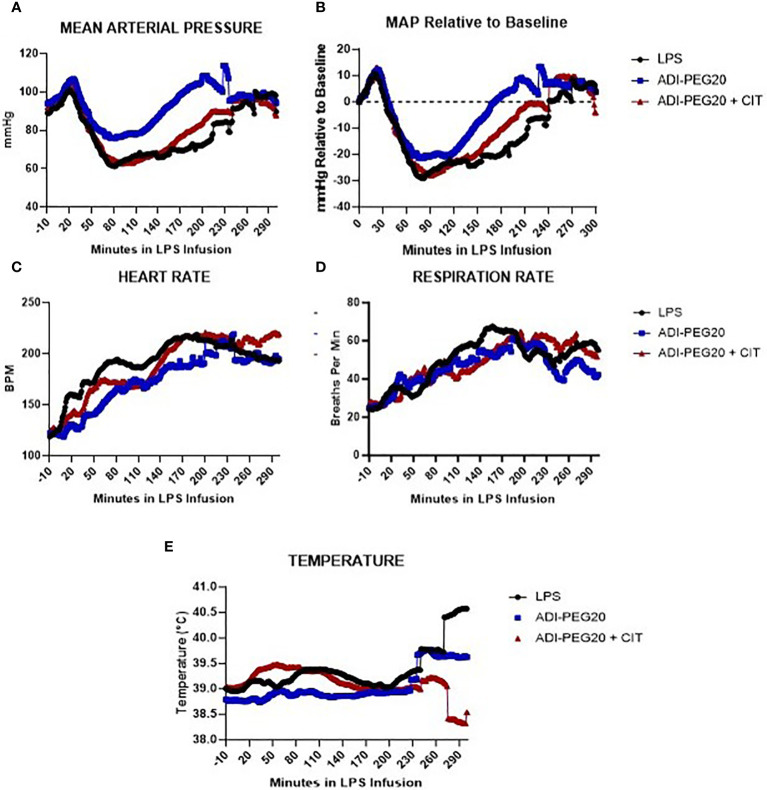
Telemetry data generated from pigs undergoing endotoxin infusion were measured using implanted probes, analyzed using repeated measures, and presented as means over time. The variation among pigs was high and is not represented on this graph. **(A, B)** Mean arterial pressure (MAP), presented as the mean in all treatments (LPS n=6; ADIPEG n=7; ADIPEG + CIT n=7), dropped (P<0.05) after 1 h of infusion but increased to a level similar to that at the beginning of the infusion by the end of the infusion. There were no statistical differences between treatments throughout the infusion (P>0.1). Heart rate **(C)** and respiration rate **(D)** were also similar among treatments (P>0.1) **(E)** and both increased throughout the infusion until hour 3 (P<0.05), at which point they remained similar until the end of the protocol. Mean temperature remained consistent across time and treatment (P>0.10).

MAP spiked over 100 mmHg 20 min after the start of the LPS infusion, then dropped to a nidus of approximately 60 mmHg and remained low for approximately 3 h. In each treatment, the pigs returned to baseline MAP (~ 80 mmHg) toward the end of the infusion (**Figures 3A, B**; *P*=0.13). Heart rate was similar (*P*=0.57) across treatments and steadily increased from the pre-infusion rate of 120 bpm to approximately 200 bpm (**Figure 3C**). Respiration rate numerically increased across treatments and was not statistically different over time (**Figure 3D**; *P*=0.73). Body temperature did not statistically change across treatments, although there was a great deal of variation among the pigs (**Figure 3E**; *P*=0.79).

**Figure 4:**
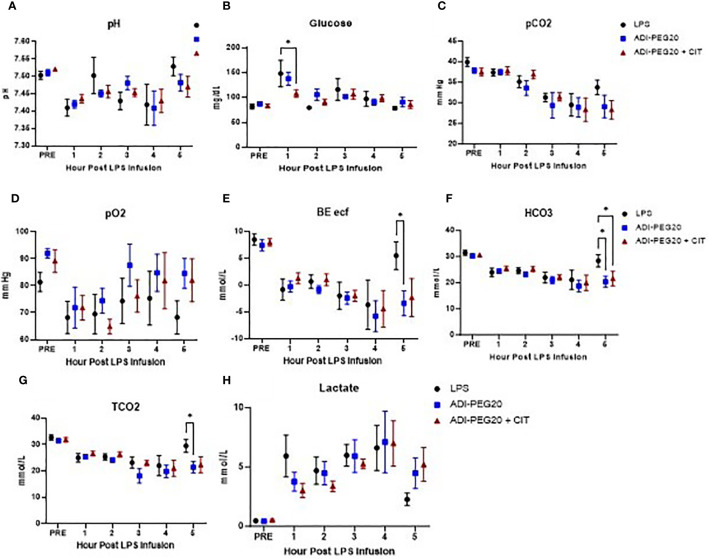
IStat clinical chemistries (CHEM8+ cartridges) were measured throughout the endotoxin infusion and are presented as mean and SEM. Blood pH **(A)** did not differ over time or across treatments (LPS n=6; ADIPEG n=7; ADIPEG + CIT n=7) (P>0.1). Blood glucose **(B)** was higher in the LPS and ADIPEG pigs than in the ADIPEG+CIT pigs at hour 1 of the endotoxin infusion (P<0.05) but did not differ across treatments at any other time point during the infusion (P>0.10). The partial pressure of carbon dioxide (pCO2; **C**) was statistically similar across treatments throughout the infusion (P>0.10). The partial pressure of oxygen (pO2; **D**) was also similar across treatments throughout the infusion (P>0.10). The base excess of the extracellular fluid (BE ECF; **E**) dropped in all treatments by hour 1 of the infusion and was higher in the LPS pigs at hour 5 than in the ADIPEG and the ADIPEG + CIT pigs (P<0.05). Bicarbonate levels (HCO3; **F**) were similar across treatments until hour 5 of the infusion, at which point pigs on the LPS treatment had higher HCO3 than ADPIPEG and ADIPEG + CIT pigs (P<0.05). Total carbon dioxide (TCO2; **G**) was also similar across treatments from hours 1–4 of the infusion (P>0.10), but was greater in the LPS pigs at hour 5 (P<0.05). Finally, blood lactate levels **(H)** increased similarly (P>0.10) across treatments from pre-infusion levels to hour 1 of the infusion and remained elevated throughout the endotoxin infusion period. *P(TRTxTime) <0.05.

Blood was sampled throughout the LPS infusion for ISTAT cage-side point of care chemistry analysis (**Figure 4**). As expected, the pH dropped in pigs 1 h after the initiation of LPS infusion in all pigs, regardless of treatment (*P*<0.05) (**Figure 4A**). Glucose was higher (*P*<0.05) in the LPS and ADI-PEG20 pigs than in the ADI-PEG20 + CIT pigs 1 h into the LPS infusion but was similar across treatments throughout the rest of the infusion; however, this may be a function of the pig’s response to exogenous glucose infusion rather than endogenous production or mobilization (**Figure 4B**). The partial pressures of carbon dioxide and oxygen (pCO2 and pO2) were similar across treatments throughout the infusion (*P*>0.05) (**Figures 4C, D**). Base excess of the extracellular fluid (BE ECF) dropped (*P*<0.05) in all pigs by 1 h post infusion but increased in the LPS pigs after 4 h of infusion. Bicarbonate (HCO3) and total carbon dioxide (TCO2) were similarly higher in the LPS pigs after 5 h of infusion, which suggests that the control pigs were starting to experience a metabolic alkalosis by the end of the LPS infusion (**Figures 4E–G**). Lactate increased from baseline in all treatments after 1 h of LPS infusion and remained high until the end of the experiment (**Figure 4H**).

**Figure 5:**
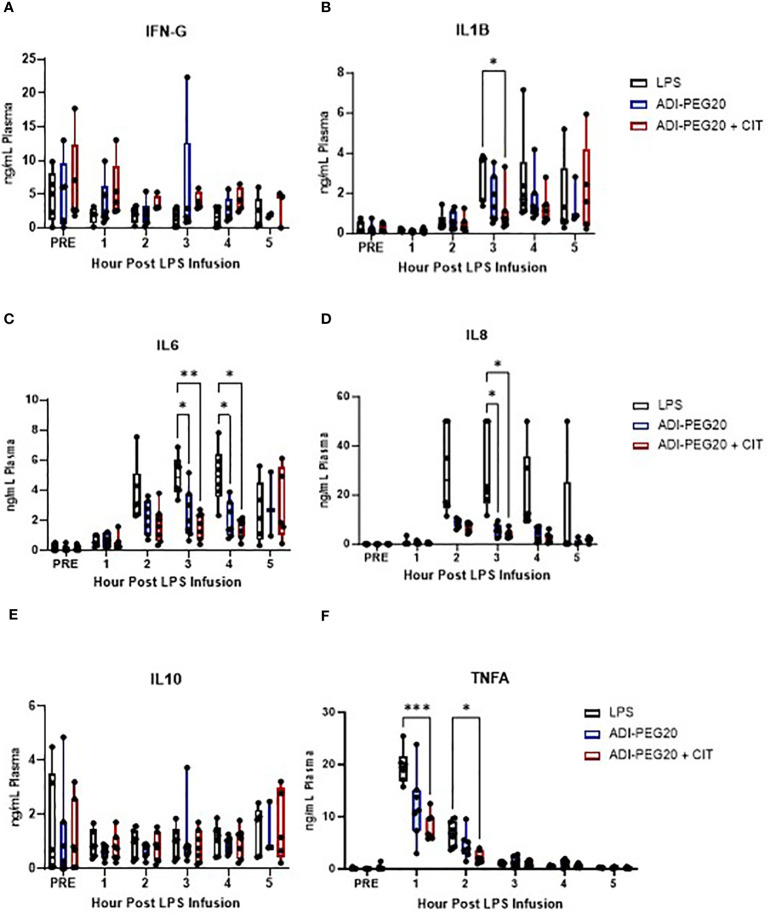
Plasma cytokine levels during the 5-h endotoxin infusion, presented as mean with standard deviation and range, measured using a porcine-specific multiplex ELISA, and analyzed using a two-way ANOVA with fixed effects of treatment and time. Interferon-gamma levels (IFNG; **A**) were similar across treatments throughout the infusion (LPS n=6; ADIPEG n=7; ADIPEG + CIT n=7). Interleukin 1-beta (IL1B; **B**) was lower in the ADIPEG + CIT pigs than in the LPS pigs at hour 3 of the infusion. Interleukin-6 (IL-6; **C**) was higher in the LPS pigs than in the ADIPEG and ADIPEG + CIT pigs at hours 3 and 4 of the infusion (P<0.05). Similarly, interleukin 8 (IL-8, **D**) was higher in the LPS pigs than in the ADIPEG and ADIPEG + CIT pigs at hour 3 of the infusion. Interleukin-10 (IL10; **E**) was similar across treatments (P>0.10) throughout the endotoxin infusion. Tumor necrosis factor-alpha (TNFA; **F**) was greater in the LPS pigs than in the ADIPEG+CIT pigs at hours 1 and 2 of the infusion (P>0.05) but were otherwise similar across treatments. *P(TRTxTime) <0.05; **P(TRTxTime)<0.01; ***P(TRTxTime) <0.001.

Pro-inflammatory cytokine levels were measured in pigs throughout the LPS infusion, and some were impacted by CIT and ADI-PEG20 treatment. There were no statistically significant differences in IFN-G throughout the LPS infusion (P>0.05) (**Figure 5A**). IL1-B was lowest in ADI-PEG20 + CIT pigs at hour 3, although was even lower at hour 4 of the infusion but not statistically significantly so (**Figure 5B**). IL-6 was lower in ADI-PEG20 and ADI-PEG20 + CIT pigs than in the control at hours 3 and 4 (**Figure 5C**). IL-8 tended to be lower in ADI-PEG20 (*P*=0.06) and ADI-PEG20 + CIT (*P*=0.07*)* pigs at hours 2 and 4 and significantly lower at hour 3 (**Figure 5D**). IL-10 was not statistically different across treatments or over time (**Figure 5E**). Additionally, IFN-G, IL10, and TNFA levels returned to pre-infusion levels by the end of the infusion (**Figure 5F**).

**Figure 6:**
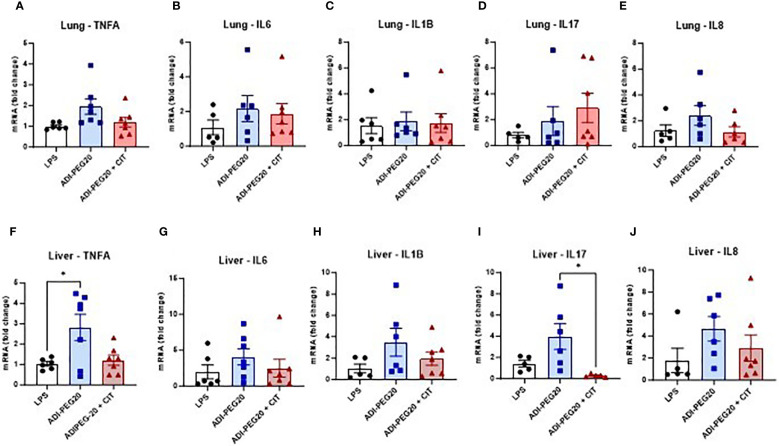
Relative expression of tumor necrosis factor-alpha (TNFA), interleukin-6 (IL6), interleukin 1-beta (IL1B), interleukin 17 (IL17), and interkeukin 8 (IL8) in the lung **(A–E)** and liver **(F-J)**, measured using RT-qPCR and analyzed using one-way ANOVA. Values are represented as mean ± SEM. *P(TRT) <0.05.

There were no differences observed across treatments in the relative expression of TNFA, IL6, IL1B, IL17, and IL8 in the lungs (P>0.05) (**Figures 6A–E**). Additionally, no statistical difference was observed in the expression of TNFA, IL6, IL1B, IL17, and IL8 in the kidney (P>0.05) (**Supplementary Figures 1A–E**). IL1B was more highly expressed (*P*<0.05) in the small intestines of ADI-PEG20 pigs. Additionally, there was similar expression of TNFA, IL6, IL17, and IL8 in the small intestine (**Supplementary Figures 1F–J**). TNFA was more highly expressed (P<0.05) in the livers of ADI-PEG20 pigs. Furthermore, the expression of IL17 was lower (*P*<0.05) in ADI-PEG20 + CIT pigs than in ADI-PEG20 pigs. No differences were observed in the expression of IL6, IL1B, or IL8 (**Figures 6F–J**).

**Figure 7:**
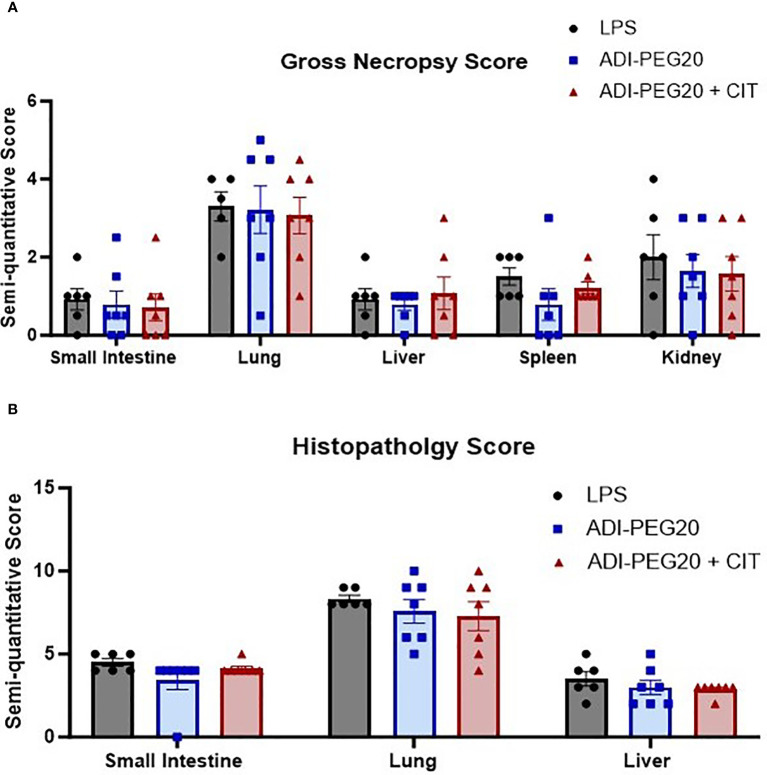
Gross and histologic injury scores of tissues after endotoxin infusion (LPS n=6; ADIPEG n=7; ADIPEG + CIT n=7). Data are represented as means +/- SEM. Gross scores **(A)** were assessed by a licensed veterinarian based on the gross appearance of the small intestine, lung, liver, spleen, and kidney. There were no differences in the mean injury score in any tissue between treatments (P>0.10). Additionally, H&E-stained slides **(B)** of the small intestine, lung and liver were scored for injury by a boarded veterinary pathologist. No statistical differences were detected between treatments (P>0.10).

**Figure 8:**
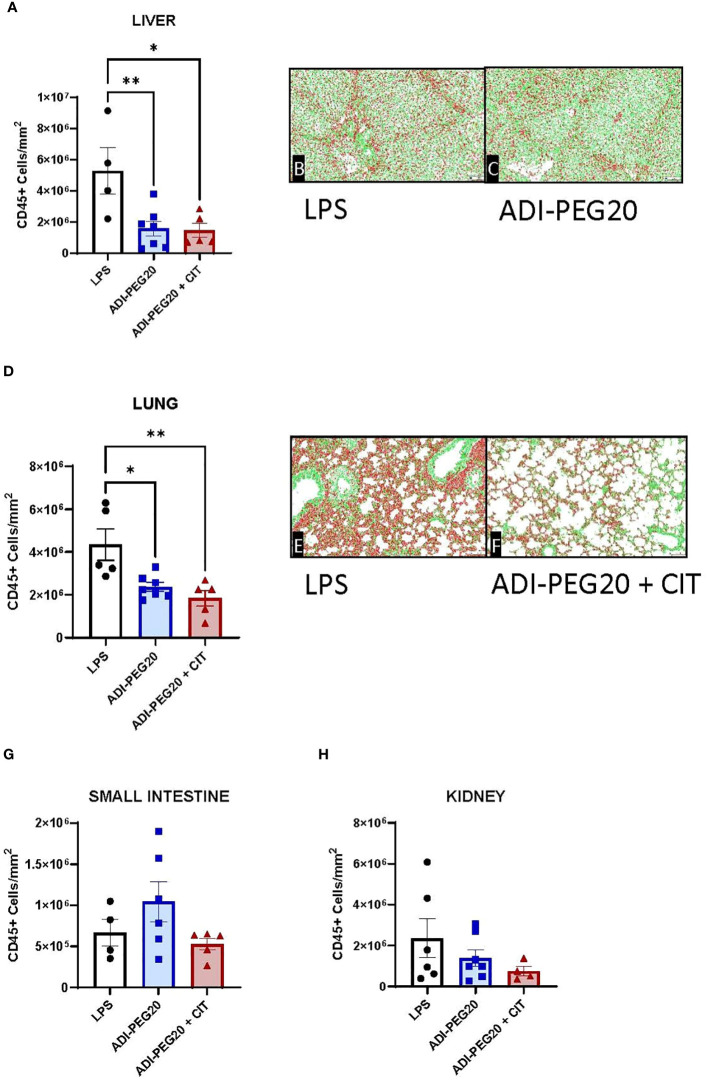
Immunohistochemical quantification of CD45+ cells in the liver, lung, small intestine, and kidney measured using a colorimetric algorithm and corrected for the number of nuclei infusion (LPS n=6; ADIPEG n=7; ADIPEG+CIT n=7). Mean CD45+ cell count is represented as ± SEM. Liver and lung CD45+ cells **(A, D)** were greater in LPS pigs than in ADIPEG and ADIPEG + CIT pigs. Panels **(B, C, E, F)** are representative images of the stained and scanned liver and lung slides with the overlying algorithm highlighting immunohistochemically stained cells (red) relative to tissue parenchyma (green). There were no statistical differences in CD45+ cell count in the small intestine and kidney **(G, H)**. *P(TRTxTime) <0.05; **P(TRTxTime)<0.01.

There were no differences between treatments (*P*>0.05) observed grossly or through histopathology (**Figures 7A, B**) after pigs underwent the endotoxin challenge. Digital quantification of CD45+ (macrophages, lymphocytes and other pro-inflammatory cells) infiltrate showed fewer CD45+ cells in the liver and lung tissue of the ADI-PEG20 and ADI-PEG20 + CIT pigs than in the control (LPS) pigs (*P*<0.05; **Figures 8A–F**). No differences in quantification of CD45+ cells were observed in the small intestine or kidney (**Figures 8G, H**).

### 4.2 Arginine metabolism

**Figure 9:**
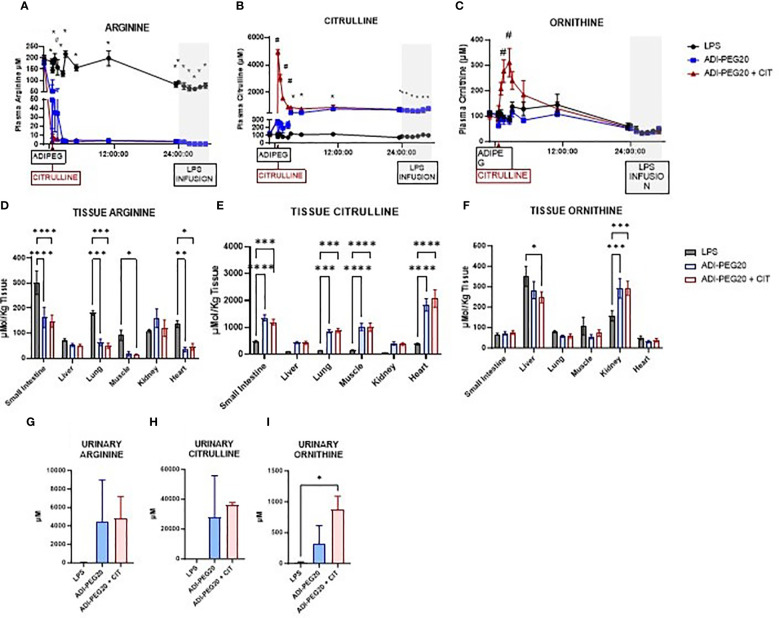
Plasma, tissue, and urinary arginine, citrulline, and ornithine are represented as means ± SEM. Plasma arginine **(A)**, citrulline **(B)**, and ornithine **(C)** were analyzed as repeated measures. *P(TRTxTIME) LPS vs. ADIPEG and ADIPEG + CIT <0.05; #P(TRTxTIME) ADIPEG + CIT vs. LPS and ADIPEG <0.05 (LPS n=6; ADIPEG n=7; ADIPEG+CIT n=7). Tissue levels of arginine **(D)**, citrulline **(E)**, and ornithine **(F)** were measured in the small intestine, liver, lung, muscle, kidney, and heart and measured as a one-way ANOVA (LPS n=6; ADIPEG n=7; ADIPEG + CIT n=7). *P(TRT) <0.05; **P(TRTxTime)<0.01; ***P(TRTxTime) <0.001 ****P(TRTxTime) <0.0001. Urinary arginine **(G)**, citrulline **(H)**, and ornithine **(I)** were measured (N=2 per TRT) and compared using Student’s T tests. *P(TRT) <0.05.

In the LPS pigs, plasma arginine concentration was higher on day one than on day two of the study period (*P* < 0.05). Plasma arginine concentration was similar immediately before and throughout the endotoxin infusion. In the ADI-PEG20 and ADI-PEG20 + CIT pigs, plasma arginine levels dropped precipitously to < 5 μM within 5 min post ADI-PEG20 injection (*P*<0.05) (**Figure 9A**). Plasma citrulline levels remained low in LPS pigs. As expected, plasma citrulline markedly increased approximately 10 fold in ADIPEG + CIT pigs immediately after intravenous citrulline bolus (*P*<0.05); however, citrulline levels decreased at 3 h post CIT infusion. Plasma citrulline in ADI-PEG20 pigs increased for 3 h post ADI-PEG injection and was similar to ADI-PEG20 + CIT pigs until the end of the study period (**Figure 9B**). Plasma ornithine was measured as a marker of arginine and citrulline metabolism and was higher (*P*<0.05) in the ADI-PEG20 + CIT pigs than in the LPS and ADI-PEG20 pigs (**Figure 9C**). Urinary arginine, citrulline, and ornithine were numerically higher in the ADI-PEG20 and ADI-PEG20 + CIT pigs than in the controls (**Figures 9G–I**).

Arginine levels in the small intestine, lung, skeletal muscle, and heart were lower in ADI-PEG20 and ADI-PEG20 + CIT pigs than in LPS pigs. There were similar concentrations of arginine in LPS, ADI-PEG20, and ADI-PEG20 + CIT liver and kidney (**Figure 9D**). Citrulline levels were higher (*P*<0.05) in the ADI-PEG20 and ADI-PEG20+CIT pigs in the small intestine, lung, muscle, and heart, and tended to be higher (*P*<0.10) in the liver and kidney (**Figure 9E**). Ornithine levels were similar in the small intestine, lung, muscle, and heart across treatments (*P*>0.05). However, ornithine levels were lower in the liver of the ADIPEG + CIT pigs (*P*<0.05) but higher in the kidney of the ADI-PEG20, and ADI-PEG20 + CIT pigs relative to the control (LPS) (*P*<0.05; **Figure 9F**).

**Figure 10:**
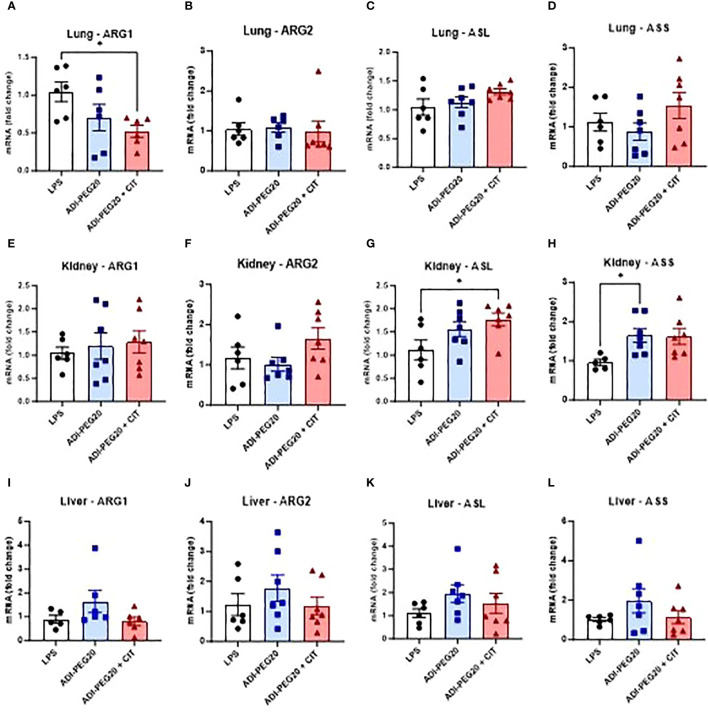
Relative mRNA expression of enzymes involved in arginine metabolism: arginase 1 (ARG1), arginase 2 (ARG2), arginosuccinate lyase (ASL), and arginosuccinate synthase (ASS) were measured using RT-qPCR (LPS n=6; ADIPEG n=7; ADIPEG+CIT n=7) in the lung **(A-D)**, kidney **(E-H)**, and liver **(I-L)**. *P(TRT) <0.05.

ARG1 expression was lower (*P*<0.05) in the lung in the ADI-PEG20 + CIT pigs (**Figure 10A**) than in the LPS and ADI-PEG20 pigs. The expression of ARG1 was not different in the liver (**Figure 10I**), kidney (**Figure 10E**), and small intestine (**Supplementary Figure 3A**). There were differences (*P*>0.05) in ARG2 expression in the lung (**Figure 10B**), liver (**Figure 10J**), kidney (**Figure 10F**), and small intestine (**Supplementary Figure 3B**). The expression of ASL was higher (*P*<0.05) in the kidney (**Figure 10G**) of the ADI-PEG20 + CIT pigs and in the small intestine (**Supplementary Figure 3C**) of the ADI-PEG20 pigs. The relative expression of ASS was higher in kidney tissue of ADI-PEG20 pigs than in control animals (**Figure 10H**). The expression of ASS and ASL did not differ across treatments in the lung (**Figures 10C, D**) or liver (**Figures 10K, L**).

## 5 Discussion

The aim of this study was to test the efficacy of ADI-PEG20 with and without supplementary intravenous citrulline to ameliorate hypotension and tissue injury in a juvenile porcine model of endotoxemia and hemodynamic shock. We hypothesized that the depletion of intravascular arginine by ADI-PEG20 limits the ability of macrophages to produce NO, thus limiting pro-inflammatory cytokines that lead to tissue injury and preventing hypovolemic shock. The use of ADI-PEG20 resulted in the depletion of arginine in small intestine, lung, muscle, and heart, while increasing CIT levels in those tissues. Our previous study in healthy pigs showed that ADI-PEG20 is an effective strategy for depleting plasma arginine with no cardiovascular effects, while simultaneously maintaining tissue arginine due to the robust local citrulline recycling capacity (53). We also showed in mice that arginine depletion by ADI-PEG20 had no effect on cardiovascular endpoints in healthy mice but limited NO production after endotoxin challenge. To expand upon these previous findings, we aimed to test whether ADI-PEG20 can deplete plasma arginine and thereby limit the pro-inflammatory NO production. We combined ADI-PEG20 with citrulline treatment to maintain NO production by microvascular endothelial cells and allow for normal cardiovascular homeostasis and mitigation of septic shock (48).

Distributive shock is a condition characterized by a failure of the circulatory system to adequately facilitate oxygen delivery to tissues. The etiology of distributive shock differs from patient to patient, but in patients with underlying sepsis or endotoxemia, septic shock is a result of a profound and dysregulated immune response (54). Pro-inflammatory cytokines and NO released by accumulating leukocytes cause central macrovascular vasodilation of the major arteries, reducing systemic vascular resistance and causing a drop in MAP. When MAP drops, this results in a loss of membrane and endothelial integrity and therefore tissue damage. Additionally, this cytokine-driven drop in MAP, coupled with the use of pharmaceutical interventions like vasopressors, triggers regional microvascular vasoconstriction in tissues such as the gut and kidney (54). ADI-PEG20 depletes intravascular arginine with the intention of reducing the capacity for macrophages to produce intraluminal NO, while simultaneously producing citrulline that can be transported into endothelial cells. Intracellular citrulline can be used to produce arginine that can be utilized within tissues or converted to NO to maintain microvascular vessel patency and preserve regional perfusion to the kidney and small intestine.

Our results showed clinical evidence of shock based on evidence of decreased MAP, blood glucose instability, and increased blood lactate, but MAP spontaneously recovered by hour 5 of the endotoxin infusion protocol. Our clinical chemistry measures support the diagnosis of acute sepsis based on reduced pH and oxygenation across treatments for hours 2–4 during the infusion, which then increased by hour 4. Additionally, the increases in proinflammatory cytokine (IL1B, IL6, and IL8) concentrations suggest that endotoxin infusion led to severe inflammation consistent with previous reports in young pigs (55). By contrast, the plasma levels of these proinflammatory cytokines were lower in the pigs treated with ADI-PEG20 and ADI-PEG20 + CIT, suggesting a dampening of inflammation induced by LPS. However, these changes in response to LPS or LPS + ADI-PEG20 and ADI-PEG20 + CIT were not observed in proinflammatory gene expression in key tissues.

Lungs react differently to other organs to endotoxin because they do not exhibit the same degree of endotoxin tolerance as other cell types (56). It has been suggested that previous exposure to endotoxin primes alveolar macrophages to have a greater activation of TLR-2 and TLR-4 and a greater expression of IL1-beta and IL-6 during a subsequent challenge than cells that were not previously exposed to endotoxin (57). *Ex vivo* analysis of rodent-derived macrophages from the spleen, peritoneum, and bone marrow show that these cells develop endotoxin tolerance, demonstrating alterations in NFKB signaling, whereas pulmonary macrophages (58). This phenomenon may explain the significant pulmonary inflammatory infiltrate observed in this study that was alleviated by ADI-PEG20.

We also examined various markers of tissue injury based on histological and immunohistological measures. We found no differences in gross or histologic scoring in any organs. This may be a function of endotoxin tolerance, the brevity of the infusion protocol, or the less-sensitive histopathology assessments. However, examination of tissue inflammation using immunohistochemical staining for CD45+ cells showed that pigs treated with ADI-PEG20 and ADI-PEG20 + CIT had fewer CD45+ cells in the liver and lungs than control (LPS) animals. However, we did not observe differences in CD45+ cells in the small intestine and kidney.

We measured the concentration of arginine, citrulline, and ornithine in plasma and tissues to examine the effect of LPS infusion and efficacy of treatment with ADI-PEG2- and ADI-PEG20 + CIT. Additionally, we confirmed the efficacy of ADIPEG alone in rapidly depleting plasma arginine and markedly increasing plasma citrulline, before and after LPS infusion. Thus, it would appear that ADI-PEG20 reduced the availability of intravascular arginine for NO production by circulating macrophages. However, in contrast to our findings in healthy pigs, in LPS-infused pigs, we found that ADI-PEG20 and ADI-PEG20 + CIT treatments had differential capacities for maintaining tissue arginine content. Pigs on both ADI-PEG20 and ADI-PEG20 + CIT treatments maintained tissue arginine content in liver and kidney, but like plasma arginine, it was markedly depleted in the lung, small intestine, heart, and skeletal muscle tissue. This latter observation was despite the fact that tissue citrulline content was significantly increased in all tissues.

The expression of genes associated with arginine synthesis (ASS and ASL) and catabolism (ARG1 and ARG2) was measured to assess the impact of ADI-PEG20 with and without supplementary citrulline on arginine metabolism in the lung, liver, small intestine, and kidney. The expression of ARG1 was lower in the lung in ADI-PEG20 pigs, and this was driven by the lower number of macrophages (suggested by the CD45+ staining) in the lungs. There were no statistically significant differences across treatments in the expression of ARG2 in the lung, liver, kidney or small intestine. ASL mRNA expression was higher in the kidney of ADI-PEG20+CIT pigs than in the kidney of LPS pigs. Additionally, ASS expression was higher in the kidney of ADI-PEG20 pigs. This suggests that the kidneys in ADI-PEG20-treated animals maintained local tissue arginine levels by increasing the transcription of ASS and ASL.

El-Awady et al. showed that using agmatine to block iNOS expression attenuated vascular hyporeactivity and endothelial dysfunction in rodent models (59). However, blocking the activity of iNOS or using generalized NO inhibitors has not improved clinical outcomes in human patients and may worsen hemodynamics in critically ill patients (60). These data suggest that there are fundamental species differences that limit the translational utility of rodent models for sepsis, specifically with regard to arginine and NO metabolism. Nevertheless, the selective blockage of NO production in septic human patients has been successful. Methylene blue semi-selectively blocks the activity of iNOS and successfully reduced renal tubular damage in adult patients with bacterial sepsis (61). Other investigators have shown that the use of a specific NOS-1 inhibitor can successfully and safely mediate changes in blood flow in healthy volunteers and there is an intention to use this intervention in future clinical trials with septic patients (62).

Although we acknowledge that the most clinically relevant approach to this experiment would have been to administer the ADI-PEG20 and citrulline treatments at the time of sepsis induction, this study was designed to assess the potential mechanistic benefits of intravascular arginine depletion before a hyperinflammatory stimulus. Future experiments will assess the efficacy of ADI-PEG20 as a therapeutic intervention with sepsis and sepsis-like conditions. Overall, these data demonstrate that ADI-PEG20 shows potential as a therapy for reducing inflammation and inflammatory infiltrates in a septic porcine model, but further work is necessary to further understand the mechanisms and implications of its use in a clinical setting.

## Data availability statement

The raw data supporting the conclusions of this article will be made available by the authors, without undue reservation.

## Ethics statement

The animal study was approved by Baylor College of Medicine Institutional Animal Care and Use Committee. The study was conducted in accordance with the local legislation and institutional requirements.

## Author contributions

CV: Data curation, Formal analysis, Investigation, Methodology, Project administration, Validation, Visualization, Writing – original draft, Writing – review & editing. BS: Investigation, Methodology, Project administration, Supervision, Validation, Visualization, Writing – review & editing, Writing – original draft. ID: Formal analysis, Investigation, Methodology, Project administration, Resources, Validation, Writing – review & editing, Writing – original draft. MM: Methodology, Project administration, Writing – review & editing, Investigation, Writing – original draft. TN: Methodology, Project administration, Writing – review & editing, Resources. YJ: Formal analysis, Methodology, Project administration, Writing – original draft. MC: Funding acquisition, Project administration, Resources, Writing – review & editing. JM: Funding acquisition, Investigation, Methodology, Resources, Supervision, Writing – original draft, Writing – review & editing, Data curation, Project administration, Validation. DB: Conceptualization, Formal analysis, Funding acquisition, Investigation, Methodology, Resources, Supervision, Writing – original draft, Writing – review & editing.

## Supplementary material

Supplementary Figure 1 Relative mRNA expression of tumor necrosis factor alpha (TNFA), interleukin-6 (IL6), interleukin 1-beta (IL1B), interleukin-17 (IL17), and interleukin 8 (IL8) in the kidney **(A-E)** and small intestine **(F-J)** (LPS n=6; ADIPEG n=7; ADIPEG+CIT n=7). *P(TRT) <0.05.

Supplementary Figure 2 Concentration of lysine **(A)**, phenylalanine **(B)**, tyrosine **(C)**, isoleucine **(D)**, and leucine **(E)** in tissues (LPS n=6; ADIPEG n=7; ADIPEG+CIT n=7), and the concentration of phenylalanine and tyrosine in urine (n=2 per TRT).

Supplementary Figure 3 Relative expression of arginase 1 (ARG1; **A**), arginase 2 (ARG2; **B**), arginosuccinate lyase (ASL; **C**), and arginosuccinate synthase (ASS; **D**) presented as mean ± SEM (LPS n=6; ADIPEG n=7; ADIPEG+CIT n=7). *P(TRT) <0.05.
