# Supplementary figures and images for: Citrulline and ADI-PEG20 reduce inflammation in a juvenile porcine model of acute endotoxemia
Source: Front Immunol. 2024 Aug 8;15:1400574. doi: 10.3389/fimmu.2024.1400574 (PMC11338849; doi:10.3389/fimmu.2024.1400574)

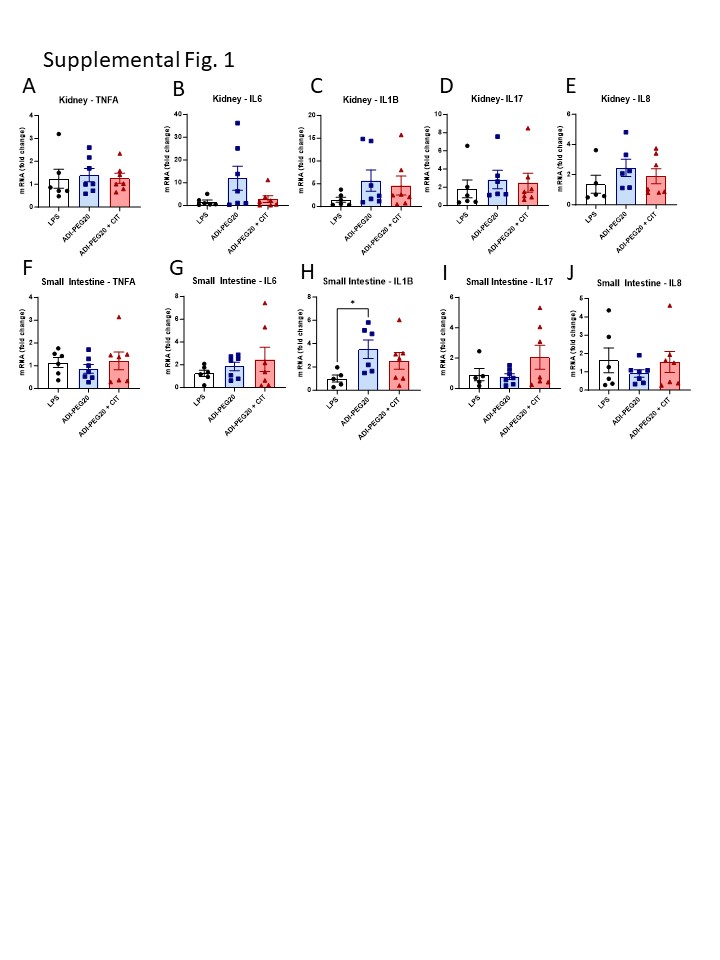

Supplement: Supplementary Figure 1 — Relative mRNA expression of tumor necrosis factor alpha (TNFA), interleukin-6 (IL6), interleukin 1-beta (IL1B), interleukin-17 (IL17), and interleukin 8 (IL8) in the kidney (A-E) and small intestine (F-J) (LPS n=6; ADIPEG n=7; ADIPEG+CIT n=7). *P(TRT) <0.05. [file Image_1.jpeg]

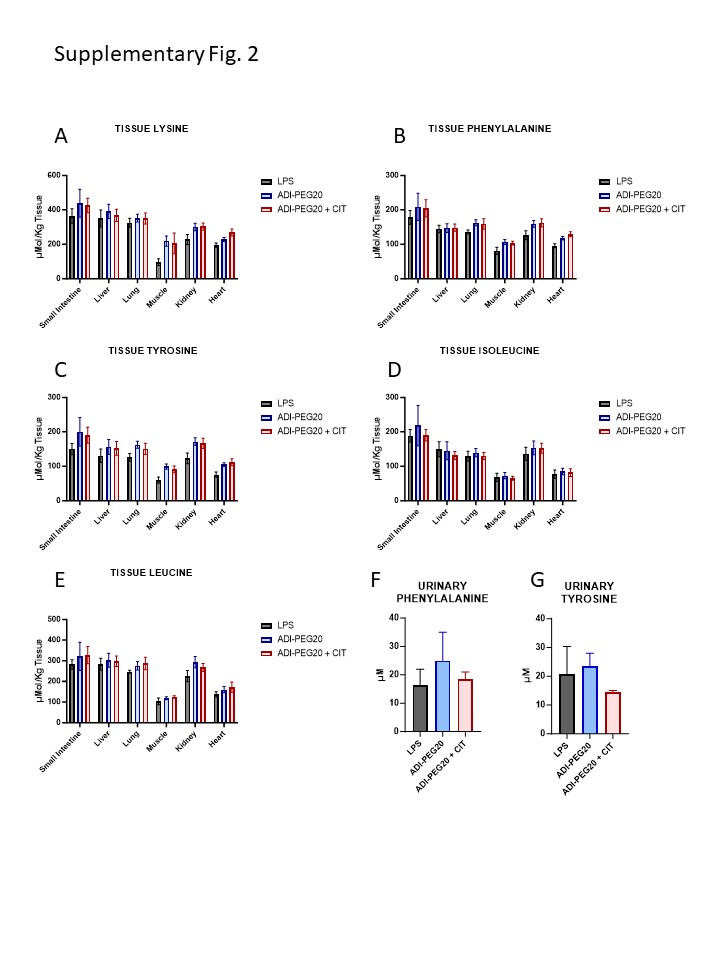

Supplement: Supplementary Figure 2 — Concentration of lysine (A), phenylalanine (B), tyrosine (C), isoleucine (D), and leucine (E) in tissues (LPS n=6; ADIPEG n=7; ADIPEG+CIT n=7), and the concentration of phenylalanine and tyrosine in urine (n=2 per TRT). [file Image_2.jpeg]

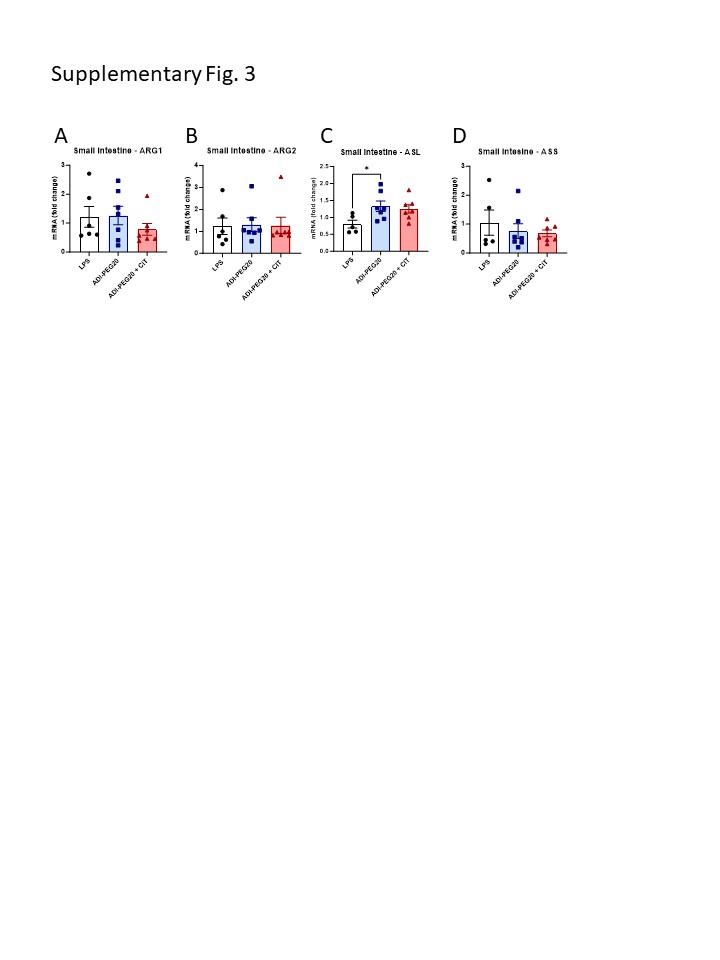

Supplement: Supplementary Figure 3 — Relative expression of arginase 1 (ARG1; A), arginase 2 (ARG2; B), arginosuccinate lyase (ASL; C), and arginosuccinate synthase (ASS; D) presented as mean ± SEM (LPS n=6; ADIPEG n=7; ADIPEG+CIT n=7). *P(TRT) <0.05. [file Image_3.jpeg]
